# NSP4 as adjuvant for immunogenicity and design of effective therapeutic HPV16 E6/E7/L1 DNA vaccine in tumor-bearing and healthy C57BL/6 mice

**DOI:** 10.1186/s13104-023-06445-5

**Published:** 2023-08-07

**Authors:** Sahar Sadr-Momtaz, Maryam Aftabi, Emad Behboudi, Malihe Naderi, Anahita Hashemzadeh-Omran, Abdolvahab Moradi

**Affiliations:** 1 Department of Microbiology, Golestan University of Medical Sciences, Gorgan, Iran; 2 Department of Basic Sciences, Khoy University of Medical Sciences, Khoy, Iran; 3 Infectious Disease Research Center, Golestan University of Medical Sciences, Gorgan, Iran

**Keywords:** Vaccine, HPV, E6/E7/L1, Adjuvant, NSP4

## Abstract

**Introduction:**

In humans, approximately 5% of all cancers are attributable to HPV infection. Prophylactic vaccines can inhibit viral migration and persistence. However, further studies are still required to develop such treatments. To achieve this goal, we designed a therapeutic HPV DNA vaccine encoding a construct of E6/E7/L1 and used NSP4 antigen as an adjuvant to assess the efficiency of this construct in generating antigen-specific antitumor immune responses.

**Materials and methods:**

Sixty female C57BL/6 mice (6–8 weeks old) were purchased from the Institute Pasteur of Iran. Through a subcutaneous (s.c) injection of a suspension of 100 µl PBS containing 10^6^ TC-1 cells/mouse in the back side, 30 of them became cancerous, while 30 of them were healthy control mice. To amplify E6/E7/L1-pcDNA3 and NSP4-pcDNA3, the competent cells of DH5α and to generate a tumor, TC-1 cell line was used. Mice were then immunized with the HPV DNA vaccine. Cell proliferation was assessed by MTT assay. Finally, cytokine responses (IL-4, IL-12, IFN- γ) were measured in the supernatant of mice spleen cells.

**Result:**

Mice receiving the NSP4/E6-E7-L1 vaccine had the highest stimulatory index compared to other groups, although it was not statistically significant. Interleukin 4/12 and IFN-γ production were significantly higher in E6-E7-L1 / NSP4 group and E6-E7-L1 group compared to other groups (P < 0.05). Among different groups, E6/E7/L1 + NSP4 group was able to slow down the tumor growth process, but it was not significant (p > 0.05). Among the aforementioned cytokines, IFN-γ and IL-12 are among the cytokines that stimulate the Th1 pathway and IL-4 cytokine stimulates the Th2 pathway and B lymphocytes.

**Conclusion:**

Our data revealed that the present vaccine can reduce tumor size, and cytokine measurement showed that it stimulates innate and acquired immune responses, thus it can be a therapeutic vaccine in the tumor-bearing mice model.

## Introduction

Human papillomavirus (HPV) is a major etiological pathogen in cancers, such as cervical, vulvar, vaginal, anal, penile, and head and neck tumors. Cervical cancer immunotherapy must target high-risk HPV16, 18 that cause 50% and 20% of cervical cancers, respectively [1, 2]. Prophylactic vaccines can inhibit HPV migration and persistence, but HPV-induced cancer is still the reason for more than 200,000 deaths in populations annually. Therefore, the need to develop such therapeutic strategies still prevails [3, 4].

The best therapeutic HPV vaccine should be capable of both eliminating virions and transforming cells expressing viral proteins. The viral oncoproteins (E6/E7) are appropriate antigen targets for immunotherapy because they are key factors in cell transformation, and they are constantly expressed by HPV-related neoplastic epithelial and tumor cells (4). The cellular immune response is responsible for removing HPV-transformed cells. Thus, optimal therapeutic vaccines should be designed in a way to raise adaptive response. Several experimental therapeutic vaccines for HPV-related oncogenesis have been introduced in trials but the majority of them had only partial signs of progress. These models were adjuvanted proteins, DNA vaccines, live vaccines, or dendritic cell-related vaccines [3].

Nevertheless, prophylactic HPV vaccines are designed to avoid the spread of HPV infection by triggering a considerable antiviral antibody response toward viral L proteins. Today two approved preventive HPV vaccines are available in the market (Cervarix and Gardasil), and their efficacy in decreasing the incidence of high-level cervical abnormalities has been confirmed [5]. Noticeably, however, due to the integration of the HPV genome in the host genome, the L1 and L2 proteins cannot be expressed in transformed cells in some stages of the disease, rendering the current vaccines useless in that phase of infection [2, 6].

On the other hand, DNA vaccines are capable of triggering the immune response by multiple mechanisms at the same time, instantly through designing several target antigens or combining immune-modulatory factors [7]. Furthermore, using these vaccines is highly beneficial, as they are resistant to temperature changes and are also cost-effective, making them a preferred vaccine choice worldwide. Although DNA vaccines have shown to be weak immunogenic vaccines in clinical trials, researchers have established a DNA vaccine strategy that uses a combination of codon-optimized constructs to target both humoral and cellular immunity [8, 9].

Using an adjuvant is an approach to increase the immunogenicity of these vaccines. Recently, researchers showed that Rotavirus nonstructural protein 4 (NSP4) has immune response stimulation functions [10]. Due to the NSP4 adjuvant properties for increasing the efficiency of the vaccine, we used the NSP4 coding plasmid together with E6/E7/L1 coding plasmid. Moreover, codon optimization, which was primarily applied to enhance protein expression, should also limit the possibility of recombination of the vaccine sequences with wild-type HPV viruses [8]. In this study, we designed a therapeutic HPV DNA vaccine encoding a construct of E6/E7/L1 and used NSP4 antigen as an adjuvant in C57BL/6 mice. Furthermore, we assessed the efficiency of this construct in generating antigen-specific antitumor immune responses to protect mice from tumor challenges and to treat preexisting tumors in mice. Finally, we determined the immunity type that is responsible for the observed antitumor responses in vaccinated, tumor-challenged mice.

## Material & methods

### Bacterial strain and cell line

The DH5α strain of Escherichia coli competent cells was used as a bacterial host for transformation in the presence of calcium chloride and heat shock treatment, based on the method of Cohen et *al* [11]. Briefly, E. coli strain DH5α was cultured at 37 °C in Luria Bertani (LB) medium, supplemented with 50 µg/mL ampicillin. TC-1 cell line (primary epithelial cell of the lung of C57BL / 6 mice) was cultured in Roswell Park Memorial Institute medium (RPMI) 1640 (Gibco BRL, Paisley, UK) supplemented with 2 mM/L L-glutamine, 1 mM sodium pyruvate, 100 U/mL penicillin, 100 µg/mL streptomycin, 5 × 10^− 5^ M β-mercaptoethanol, and 10% fetal bovine serum (FBS) at 70% confluency 20 h before transfection.

### DNA vaccine construction

**Fig. 1:**
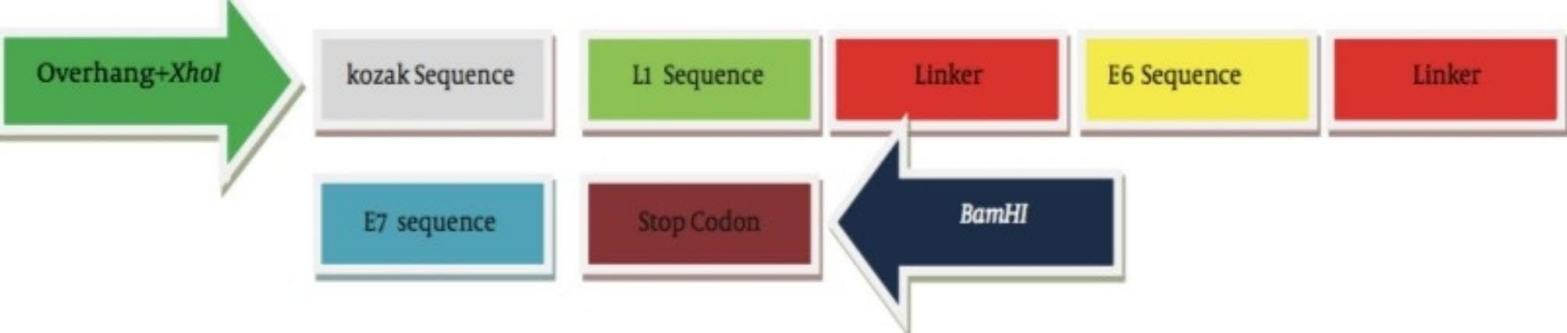
The schematic representation of the E6/E7/L1 gene expression cassette

We constructed a pcDNA3.1 plasmid containing codon optimized E6/E7/L1 gene expression cassette under human cytomegalovirus (HCMV) immediate-early promoter and provided NSP4-pcDNA3.1 expression vector from Tarbiat Modares University (Fig. 1). Purified polymerase chain reaction (PCR) products were cloned into a pcDNA3.1 cloning vector and were confirmed by sequencing. The digested gene was sub-cloned into a pcDNA3.1 eukaryotic expression vector (Invitrogen, Canada). E. coli DH5α strain bacteria were transformed with the plasmids and plated on LB plates containing 100 µg/mL ampicillin. The selected colonies were extracted using a Gene All kit according to the manufacturer’s instructions. Cloning confirmation was done by restriction enzyme digestion and PCR.

### Tumor challenge

**Fig. 2:**
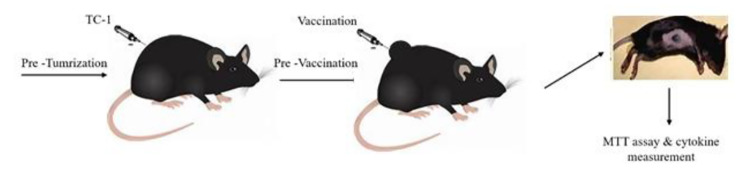
Flow diagram of experimental design

For the therapeutic experiments, C57BL/6 mice were challenged by a subcutaneous (s.c) injection of a suspension of 100 µl PBS containing 10^6^ TC-1 cells/mouse in the back side and then, were grouped into six cages. The flow diagram of the experiment was as Fig. 2.

### Vaccination

In this study, sixty female C57BL/6 mice (6–8 weeks old, weighing 17–20 g) were bought from the Institute Pasteur of Iran (Karaj, Iran). All animal experimental protocols were used according to WHO and Animal Ethics Committee of Golestan University of medical sciences approved protocols of appropriate mice care (No. Ir.goums.rec.1397.226) and the mice were acclimated with the laboratory conditions (light–dark cycles, temperatures of 22 °C ± 2 °C) for 1 week before the experiment. Mice were divided into two groups of 30, cancerous and healthy. Both groups were distributed in six sub-groups as described in Table 1.

**Table 1:** Mice groups in-experiment

Groups	Descriptions
Group I	Control (Tumor-bearing Group: TC1, Healthy Group: Sham)
Group II	PBS
Group III	pcDNA3.1+ plasmid alone
Group IV	Plasmid pcDNA3.1 + NSP4
Group V	plasmid pcDNA3.1+ E6, E7, L1
Group VI	plasmid pcDNA3.1+ E6, E7, L1 + NSP4 adjuvant

### Immunizations

For DNA vaccine immunizations, mice were immunized subcutaneously in the abdominal area with a total of 30 µg HPV DNA vaccine (HPV16-E6/E7/L1 and Rotavirus NSP4, secreted and ubiquitinated construct pooled 1:1 by weight). Mice were injected intraperitoneally with 200 µg of monoclonal antibodies targeting PD-L1 (clone B7-H1, BioXcell) one dose weekly for 3 weeks following the first vaccination.

### MTT

Two weeks after the final immunization, to euthanize mice, they were first anesthetized with ketamine (75 mg/kg) injected subcutaneously. The spleen of each mouse was removed, and lymphocyte proliferation was evaluated using the MTT method according to the manufacturer´s recommendations (Sigma) by Phytohemagglutinin (PHA) and TC1 lysate. In this way, we prepared the TC1 cell by sonicating and removing the cell wall and releasing the protein.

### ELISA

One week after the last immunization, freshly isolated splenocytes of immunized mice in the different treated groups were cultured and stimulated with PHA and TC1. The cell supernatants were collected and assayed for the presence of IFN-γ, IL-4, and IL-12 using commercially available sandwich-based enzyme-linked immunosorbent assay (ELISA) kits (in comparison to unstimulated controls) (Peprotech, New Jersey, US), following the manufacturer’s instructions. All tests were performed in triplicate for each mouse. Given the importance of vaccine immunogenicity, we chose cytokines which play the key role in defining the type of immune response. Among the cytokines mentioned, IFN-γ and IL-12 are among the cytokines that stimulate the Th1 pathway and innate immunity, and IL-4 cytokine stimulates the adaptive immunity including Th2 pathway and B lymphocytes.

### Statistical analysis

Statistical analysis was performed by Graph Pad Prism version 8.0.1. The levels of statistical significance for differences between experimental groups were determined using T-test. Survival curves were compared by a log-rank (Mantel Cox) test. Kruskal–Wallis was performed and differences were considered significant (P-value < 0.05).

## Result

### Effectiveness of vaccination on tumor size

**Fig. 3:**
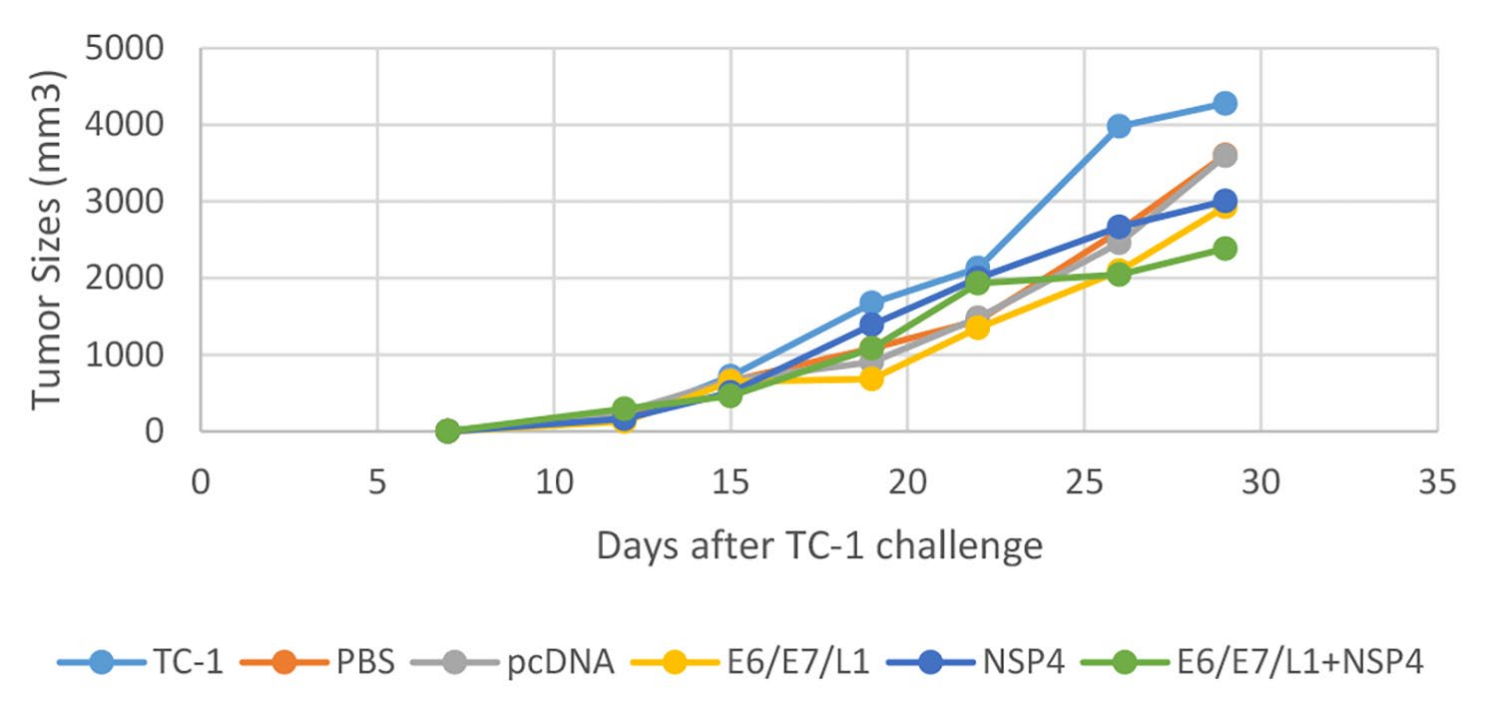
Results of tumor size measurement

Seven days after the injection of TC-1 cells, a tumor was observed in C57BL/6 mice. Tumor size was measured at 6 different time intervals during the vaccination program. The results of the measurement showed that the tumor size was lower in the vaccinated groups compared to the control groups. Interestingly, the E6/ E7/L1 + NSP4 group was able to slow down the tumor growth process compared to the other groups, but it was not statistically significant (p > 0.05). (Fig. 3)

### Lymphocyte proliferation assay

**Fig. 4:**
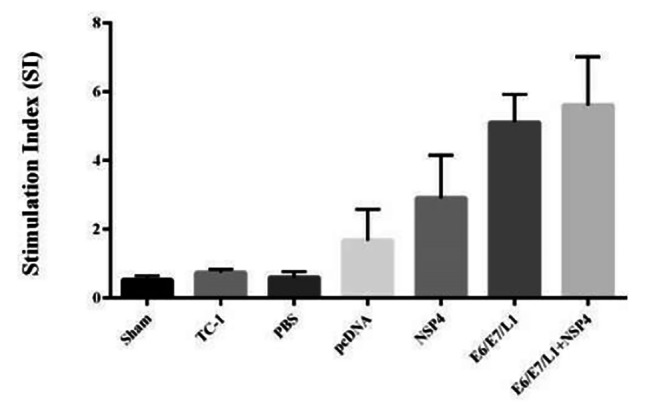
The stimulation index given by the MTT test has demonstrated that the 6th group of study has been most affected by the DNA vaccine. (Group1 control: TC1, Group2 control: Sham)

The results revealed that the stimulation index of vaccinated groups is significantly higher than the control groups (p < 0.05). The mice receiving the NSP4 / E6-E7-L1 vaccine had the highest stimulatory index compared to other groups. The comparison of the results of lymphocyte proliferation assay in different groups and the control group was not significant (p > 0.05) (Fig. 4).

### IFN- γ, IL-4 and IL-12 secretion levels

**Fig. 5:**
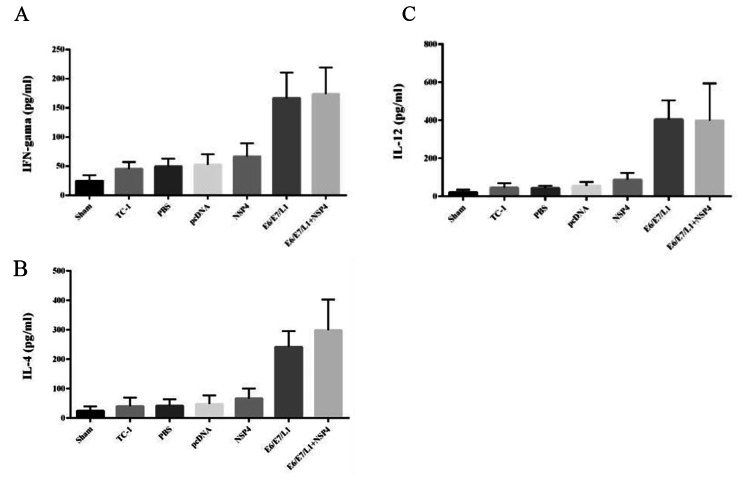
Co-administration of the NSP4 adjuvant with a E6/E/L1 vaccine induced significantly higher amounts of IFN-γ, IL-4 and IL-12 in vivo. The supernatants of splenocytes, stimulated with PHA and TC-1, from each group were collected one week after the third immunization. ELISA was performed to detect the level of IFN-γ (A), IL-4 (B), IL-12 (C) in splenocyte cultures. All of cytokines were significantly higher in E6-E7-L1 / NSP4 group and E6-E7-L1 group compared to other groups

To investigate the level of IL-4/12 and IFN-γ production, spleen cells of immunized mice in different groups were cultured and also stimulated with specific antigens. Results of nonparametric post-hoc test showed that interleukin-4/12 and IFN- γ production was significantly higher in E6-E7-L1 / NSP4 group and E6-E7-L1 group compared to control and other groups (p < 0.01) (Fig. 5). Although, the differences between these two groups (tumor-bearing and healthy) was not significant.

## Discussion

Cancer immunotherapy using DNA vaccines has emerged as a potentially promising approach for the control of tumors. This study showed that the pcDNA3.1- E6-E7-L1/NSP4 vaccine could elicit a stronger specific CD8 + T cell-mediated immune response than other groups. Our results showed that vaccination by pcDNA3.1-E6-E7-L1/NSP4 can decrease tumor size and could elicit potentially protective and therapeutic antitumor responses against HPV16 expressing TC-1 tumor model in mice. Moreover, we showed that the pcDNA3.1-E6-E7-L1/NSP4-induced antitumor immune response may be CD8 + T cell-dependent. In the current study, numerous important mechanisms contributed to the enhanced immunogenicity of pcDNA3.1- E6-E7-L1/NSP4 DNA vaccine were observed.

Previous studies reported that coadministration of DNA encoding BPVL1 enhances the immunogenicity of the E7 DNA vaccine by increasing CD4 + T cell responses that are known to assist the generation of CD8 + T cell response [12]. The CD8 + T cells served as the basis for the design of a fusion BPVL1-E7 DNA vaccine, and now we used that data for the design of an E6-E7-L1/NSP4 DNA vaccine. Additionally, several studies have tested the conjugation of E7 to L1 VLPs as a method to enhance the E7 immune response [13–15]. It has been shown that intact HPV-L1 VLPs can interact with DCs to directly induce potent adaptive immune responses in the absence of adjuvants [16]. Potentially, it occurs in a TLR-MyD88 pathway-dependent manner [17]. In contrast to our results, Tahmatan et *al*. and Sajjadian et *al*. studies showed that HPV16 DNA-E7 inoculation did not increase IL-4 expression compared to the simple vector control group [18, 19]. In Rahimi et *al.* study, there was a significant difference between using the E7 DNA vaccine and PBS. In their study, due to the simultaneous use of both Hitchner B1 and LaSota strains with the HPV-16 E7 DNA vaccine, the expression level of IL-4 was much higher than in other groups [20]. In the present study, the vaccine could also stimulate humoral immunity, which can serve as a basis for preventing future infections or treating HPV infections. Many researchers in the field of antitumor vaccines developed various vaccines for HPVs, but our study is one of the researches designing a preventive and therapeutic vaccine on mouse models which revealed the efficacy of a vaccine in cellular and humoral immunity.

The HPV E6 and E7 proteins have been demonstrated to be constantly expressed in cervical cancer cells [21]; thus they are rational targets for HPV-16 therapeutic vaccination in cases with cervical carcinoma. Studies revealed that endothelial cells are capable to take up the E7 viral protein added to the culture medium [22]. Moreover, the HPV-16 E6/E7 + cancerous cells can be genetically modulated with DNA coding for immune regulatory cytokines or adjuvants utilized for vaccination. Immunological studies have shown a key role for CD8 + T lymphocytes in the suppression of an aggressively growing carcinoma [23].

Recent studies have revealed that HPV-16 L1 protein has stimulatory effects on cellular immunity; therefore, it could be applied for the manufacture of therapeutic vaccines. Cheung et *al.* (2004) engineered the L1E7hpSCA1 vector expressing the L1 and E7 proteins with the codon optimization for mammalian cell expression. Once their vaccine was injected into the muscle, they found that this vector had strongly induced T cell cytotoxicity and also is capable of lysing the TC-1 cells expressing E7. In animal models, tumor growth was suppressed in vaccinated mice [24]. The protection toward challenge by TC-1 tumor was also revealed by vaccination with chimeric vector harboring the HPV-16 L1/L2 viral capsid proteins and the E7 oncoprotein [14]. In our study, we used E6/E7 coding genes engineered by mutating six known oncogenic regions within E6 and E7. These mutations should disable E6 to mediate the degradation of p53 and also bind to the PDZ motif inactivating the tumor suppressor protein PTPN13. Mutations in E7 (H2P, C24G, E46G, and L67R) can prevent its capability to inactivate pRb and binding to Mi2β that enhances cellular growth [25].

Since the CD8 + T cells have a key immunological role in tumor elimination, we compared in vitro cytotoxic effect of the splenic cells isolated from the different vaccinated mice. Our findings showed a rise in CTL activity of the animal vaccinated with L1, E6, and E7 as a DNA vaccine plus NSP4. For clarification of this study, we considered that improvement of E6 and E7 oncoproteins as inducers of cellular immunity accompanied by L1 and NSP4 adjuvant could be due to its specific epitopes. Bellone et *al*. (2009) found that the HPV-16 L1 DNA vaccine strongly increases the activity of vaginal CD8 + T cells, which are critical for the elimination of virus-infected cells [26].

In comparison with the Bellone et *al.* study, the findings of this study revealed that CTL response was stronger than in some previous studies, possibly because of the stage of the tumor, the cell line, the animal model, and the method of CTL assay [26]. Also, pro-inflammatory cytokines may stimulate the natural killer (NK) cells, and these NK cells have a key role in tumor growth suppression [27]. Although we didn’t evaluate NK cell activity in our animal models, the role of NK cells in tumor elimination and growth cannot be ignored. The regulation of T helper cells as effector cells is undeniable for effective immunity; hence, enhancing all immune responses is required for considerable protection [28].

## Conclusion

In summary, we developed a novel therapeutic DNA-based HPV16 vaccine, encoding an E6/E7/L1 protein and NSP4 adjuvant, which was codon-optimized for enhanced expression, and combines sequences that are secreted and ubiquitinated to induce a balanced humoral and cell-mediated immune response. We showed that this vaccine can be immunogenic and protective in the TC-1 tumor-free model and tumor-bearing mice. This approach would be one of the promising therapeutic vaccines in HPV-related cancer cases.

## Data Availability

The datasets generated during and/or analyzed during the current study are available from the corresponding author upon reasonable request.
